# From pediatric to adult care: strategic evaluation of a transition program for patients with osteogenesis imperfecta

**DOI:** 10.1186/s12913-014-0489-1

**Published:** 2014-10-31

**Authors:** Maman Joyce Dogba, Frank Rauch, Trudy Wong, Joanne Ruck, Francis H Glorieux, Christophe Bedos

**Affiliations:** Shriners Hospital for Children, 1529 Cedar Avenue, Montreal, Quebec, QC H3G 1A6 Canada; Department of Family and Emergency Medicine, Laval University, 1050 Medicine Avenue, Quebec, G1V 0A6 Canada; Faculty of Dentistry, McGill University, 3550 University Street, Montreal, QC H3A 2A7 Canada; Department of Social and Preventive Medicine, Faculty of Medicine, Université de Montréal, C.P. 6128, Succ. Centre-Ville, Montreal, QC H3C 3 J7 Canada

**Keywords:** Transition program, Osteogenesis imperfecta, Program evaluation, Healthcare system, Child and adolescent health

## Abstract

**Background:**

Achieving a successful transition from pediatric to adult care for young adults with special needs, especially rare genetic diseases such as osteogenesis imperfecta (OI), is a prominent issue in healthcare research. This transition represents a challenge for patients with OI, their families, clinicians and healthcare managers because of the complex nature of the process and the lack of evaluation of existing transition programs. We evaluated a transition program for adolescents and young adults with OI from a pediatric orthopedic hospital to adult care.

**Methods:**

Data were collected by interview, observation, and document review from April 2013 to October 2013. Participants included six patients with OI, four parents, and 15 staff, including administrators, coordinators, social workers, nurses, pediatricians, surgeons, occupational therapists and physiotherapists. A SWOT (Strengths, Weaknesses, Opportunities and Threats) analysis was performed.

**Results:**

The strengths of the transition program included a solid theoretical approach based on a partnership with parents, and a comprehensive transition model based on fostering independent living and professional integration. The program’s main weaknesses were the successive organizational changes and discontinuation of certain transition activities, and the potential conflict between the transition program and participation in research protocols. Further opportunities include the implementation of a multi-site transition model with cross-site personnel and user evaluations, with the inclusion of second-generation patients. Dissatisfaction reported by some care-team members at the adult care hospital could threaten collaboration among institutions involved in the transition process, whereas dissatisfaction of some former patients may reduce their perceptions of quality of care received during the transition.

**Conclusions:**

This study confirmed that a “one-size-fits-all” transition model for patients with OI would be inappropriate across, or even within institutions. Opportunities should be seized to create tailored, theoretically-sound transition programs that reflect patient preferences, especially those of young adults with complex and chronic health conditions. Alignment with other organizational activities should be considered, and ongoing evaluation of transition programming may be required. This SWOT analysis and utilization-focused evaluation has led to a comprehensive new project to improve the transition program for patients with OI and other conditions requiring special follow-up.

## Background

Achieving a successful transition from pediatric to adult care has become a prominent topic in health care research. It is estimated that at least 12% of adolescents (aged 10–19 years) and young adults (aged 20–24 years) live with a congenital or acquired chronic condition [1]. These individuals need effective transition programs that empower them to manage their health independently during adulthood. Poor transition programs may result in increased morbidity, increased hospitalizations, decreased hospital attendance, and discontinuity of care [2]. Young adults with special needs are particularly vulnerable, especially those with rare genetic diseases accompanied by some level of disability [3,4], such as osteogenesis imperfecta (OI), also known as brittle bone disease.

OI is a rare genetic disorder that causes increased bone fragility and reduced bone mass [5,6]. There are seven recognized clinically-defined types of OI (types I–VII), though other genetically-defined types have also been described [5]. Apart from fractures, OI can be associated with short stature, limb and spine deformities, and restricted mobility. Mild forms of OI generally have a non-progressive course and are therefore associated with normal life expectancy. However, in the severe forms (type III), life expectancy is decreased [7]. Although the course of OI is characterized by a decrease in fracture incidence over time, patients with OI still need regular check-ups to detect other age-related impairments (e.g., hearing, cardiac or pulmonary disorders). Moreover, reduced mobility and dysmorphisms may impact on the social lives of adolescents with OI and their sense of self-efficacy [8]. Young adults affected by severe forms of OI have also expressed a need to be supported in terms of reproductive issues, such as whether or not they are able to/should have children [9]. Both medical and non-medical care are therefore needed during the transition from pediatric to adult healthcare for patients with OI.

Despite the complex issues and challenges surrounding the transition from pediatric to adult care for patients with OI and their families, clinicians, and healthcare managers, there is currently a lack of evaluative research on existing transition programs [10,11]. Research to date has focused on identifying effective transition models and creating recommendations for successful transitional care. However, in this era of evidence-based practice, formal evaluations of transitional care are required [12].

Here we report on the evaluation [13] of a program that aims to help adolescents with OI in the transition from pediatric to adult care. We assessed gaps between the initial program plan and program delivery with the aim of identifying the program’s strengths and weaknesses and making recommendations for its improvement. We specifically aimed to identify the strengths, weaknesses, opportunities and threats to the Shriners Hospital for Children (SHC) transition program from the perspectives of the services users and staff.

## Methods

### The transition program

#### Context

The SHC in Montreal (Canada) is part of a network of 22 pediatric hospitals located throughout North America [14]. The SHC provides integrated and multidisciplinary complex medical care to children with musculoskeletal disorders (including OI). In 2013, this hospital provided care for 472 children and adolescents with OI, including 265 (56%) from Canada, 142 (30%) from the United States and 65 (14%) from other countries. Among the Canadian patients, 154 (58%) were from the province of Quebec, where the hospital is located. The different origins of patients treated at the SHC may thus pose challenges in terms of harmonizing transition procedures because of differences in healthcare systems. For example, the upper age limit for attending most pediatric hospitals in Canada and the United States is 18 years, while the SHC allows treatment and follow-up for patients with rare diseases until the age of 21. Furthermore, differences in health insurance coverage mean that some young adults with OI do not have access to the same services in their local healthcare institutions, whereas all medical treatments, including drugs, surgeries, rehabilitation and braces, are provided with no out-of-pocket expenses to patients within the SHC system. Furthermore, the SHC’s mandate means that most patients, especially those who live far away, are eligible for funding to cover the costs of transportation, lodging and meals [14]. Finally, access to social and support services depend on the extent of the patient’s disability, but also on the specific privileges granted in different provinces and states. Currently, participation of patients from the SHC in this program is sponsored by SHC philanthropic donors.

#### The transition model

In 2002, the SHC implemented family-centered, transition-focused care for its patients with OI. This model incorporates the transition from pediatric to adult care into the overall health care coordination system, conceptualizing care coordination as a clinical process that enhances transition outcomes. This system-wide program was designed to provide holistic and comprehensive healthcare to all SHC patients, and to incorporate transition within the all-encompassing care delivered by the care coordination services. According to the SHC model, the overlapping, inter-related care coordination and transition services share a common set of goals, outcomes, and processes. These services begin at entry into the SHC system. They are gradual, individualized, and designed to assist children and youths to: i) achieve optimal levels of health, independence, and self-determination; ii) live inclusively in their homes, schools and communities; and iii) prepare for the future. Within the care coordination process, each patient has a designated care coordinator who facilitates care and service. Shortly before their 14^th^ birthday, the child is referred to a transition coordinator who reviews the patient’s needs and goals. Regular meetings between the patient/family, care coordinator, and transition coordinator aim to address transition-related issues such as employment, education, living, and social and community life.

The model acknowledges that multiple transitions need to be negotiated successfully across the care continuum. These include: i) transitions as children enter and exit the SHC system; ii) developmental transitions across infancy, early childhood, school age and adolescent years; and iii) medical/health transitions with changes in health status, health care management strategies or need for resources and services. More intensive care coordination is often needed during these periods of transition, particularly during transition from SHC to adult systems of care [15,16].

#### Structure of the transition program

The transition program is managed by a Transition Committee comprising three nurses, an occupational therapist, a dietician, a physiotherapist and a social worker, appointed by the Nursing and Patient Care Services. The Committee is responsible for administering the program, but all final decisions are made by the Nursing and Patient Care Services. The Transition Committee is only responsible for care when the patient is at Shriners and does not interfere with the subsequent adult services.

#### Target population of the transition program

The transition program is designed for adolescents and young adults aged 14–21 years with a diagnosis of OI, who are followed-up at the SHC. The target population for our study period (January–November 2013) was 231 patients. Thirty of these patients were aged 21 years and were thus expected to have transitioned from the SHC by the end of 2013.

#### How the transition program works

When adolescents approach their 14^th^ birthday, they complete a transition referral form including information allowing the health professionals to identify the patients’ needs for specific care, such as dietary counseling, physical therapy or genetic counseling, and their needs for support in everyday life activities, such as obtaining a driver’s license and education. The patients are asked to request a summary of their medical records at ages 18 and 21. At their last visit to the pediatric hospital, patients are given a wallet-size card with contact information, so that future adult healthcare providers can consult with the OI team in the pediatric hospital as needed. At the age of 21, patients are referred to their local care institutions. Patients from Quebec, especially those from Montreal, often receive follow-up at the McGill University Health Centre, thanks to an agreement between a group of adult care physicians and the SHC. Under this agreement, an internist, rheumatologist, and orthopedic surgeon manage the care of adult patients with OI after they leave the SHC. Patients are requested to make the appointments with these professionals when needed. The internist usually provides systematic annual follow-ups. Since 2002, 70 patients with OI have enrolled in the transition program (30 from 2002–2005, 12 from 2006–2009, 16 from 2010–2012 and 12 during the study period [January–November 2013]).

In addition to care coordination, the transition program has also been involved with other rehabilitation centers in Quebec, including through organized social events, such as camps and get-togethers, to help young adults with special needs lead normal lives, make new friends and prepare for adult life. Young adults aged 16–25 years with a high potential for independent living and high levels of motivation to improve their social lives, as assessed by the Transition Committee, were eligible to participate in these camps. The camps were primarily instructed by francophones, and patients who were fluent in French were therefore given priority. Camp activities included adapted sports (waterskiing, archery, basketball, tennis, soccer, kayaking), grocery shopping and preparing a simple meal, activities to promote self-esteem and personal growth, community visits from local police to talk about safety, job searches and résumé writing, driving and alternative modes of transportation. Camp workshops were provided by nurses, social workers, occupational therapists, doctors, nutritionists, vocational counselors and recreational therapists. A social worker and nurses from the SHC participated in four previous camps. The full cost for this 2-week “sleep-away camp” is currently $2,750, but the SHC funded up to 96% of the cost, and most of the 14 patients who participated during the period 2009–2013 only paid about $100 each. Patients are expected to take full control of their health, and parents are therefore encouraged to let their adolescents gradually manage their own appointments and, if possible, attend the final hospital appointments alone. Social services and psychologists are available to help parents through this “letting go” process.

#### Expected outcomes of the transition program

The program is individually tailored to patient needs and conceptualizes the transition process as gradual, and extending over many years. Its goals are to assist children and youths with OI: 1) to achieve optimal levels of health, independence and self-determination; 2) to live independently inclusively in their homes, schools and the community; and 3) to prepare for the future.

### Study design

We carried out a utilization-focused evaluation aimed at promoting improvements in the SHC transition program [17]. Hospital managers and policy-decision makers therefore participated in the study, as well as conducting the research. A case study approach and qualitative evaluation were chosen to obtain an in-depth assessment of the transition process [18]. We collected data from interviews, observations and documents from April 2013 to October 2013. We conducted face-to-face semi-structured interviews with a sample of patients and parents of patients (hereafter named service users) and with health care professionals and hospital managers (hereafter named staff). We developed interview grids for service users and staff, respectively, based on previous experience and a search in the literature. The service-users’ interview consisted of open questions on the following themes: a) general information on the conditions under which the diagnosis was suspected and confirmed; b) current utilization of healthcare services and reasons; c) experiences of the SHC transition program if any; and d) recommendations for improving the transition program. The staff interview included open questions on: i) demographics, educational and professional background; ii) previous roles in the transition program; iii) opinions of the current transition model; iv) areas for improvement in the transition program; and v) features to maintain in the current transition program. Table 1 provides an outline of the interview guidelines.

**Table 1:** **Interview guidelines for evaluation of transition program for osteogenesis imperfecta patients, family and staff**

**a.**	**Patients/parents**
a.	Introduction (presentation of the study, introduction of the interviewer and time allocated to read and sign the consent forms).
b.	Please tell me about yourself/your child (the interviewer will ensure that the patient talks about his/her age, education or profession, OI type, physical disability, use of wheelchair or not, living alone or with parents or friends etc.).
c.	When were you/your child diagnosed with OI?
d.	Did you/your child stop going to the SHC? When? What was your care routine at that time?
e.	For those who have left the SHC, how do you organize your/your child’s medical care since you left the Shriners (family physician, emergency care, and other medical or surgical visits)?
f.	For those who are still followed-up at the SHC, how do you organize your/your child’s medical care?
g.	What are your experiences of the transition program of the SHC?
h.	What recommendations can you offer to improve the transition program?
i.	Please share any comments or any points to discuss that we have not talked about.
j.	End of the interview.
**b.**	**Staff**
a.	Introduction (presentation of the study, introduction of the interviewer and time allocated to read and sign the consent forms).
b.	Please tell me about your educational and professional background.
c.	Please tell me about your career since you came to the SHC. When did your involvement with the transition program begin? What were your previous roles in the transition program?
d.	What do you think about the current program?
e.	In your opinion, what should be improved in the program?
f.	How has the transition program improved over time?
g.	Please share any comments or points to discuss that we have not talked about.
h.	End of the interview.

We used purposeful sampling to maximize variation in transition status of the service users [17]. Three groups of participants were targeted: i) individuals or parents of individuals who had transitioned; ii) individuals or parents of individuals who had not transitioned; and iii) individuals or parents of individuals who had transitioned but had maintained a link with the SHC because of involvement in ongoing research. Patients and parents recruited were not necessarily paired. Individuals or parents of individuals who had transitioned for at least 2 years were identified through the Transition Committee records and invited to participate. Individuals or parents of individuals who had not transitioned and those who had maintained a link with the hospital after transitioning were approached during regular hospital visits and invited to participate in the study. Eligible participants had to be fluent in English or French, and patients younger than 14 years were excluded because they had not yet been approached by the Transition Committee. All participants signed a written consent form.

Regarding staff participation, a snowball sampling technique was used by asking identified key informants to suggest other potential participants whose opinions on the transition process would be valuable [17]. The staff comprised two groups: i) healthcare personnel involved in the management of OI patients at the SHC; and ii) nurses and physicians in the adult institutions providing care to adult OI patients who were former SHC patients. Healthcare personnel at the SHC directly implicated in the management of OI patients (physicians, nurses, physiotherapists, occupational therapists) or who were associated with decisions concerning patients (social workers) were selected. The final sample size was determined by field or theoretical saturation [19]. Field saturation was reached when a participant recommended another staff member (snowball technique) who had already been interviewed. Theoretical saturation was reached when participants’ responses became repetitive. All 15 staff targeted were recruited and theoretical saturation was reached at this point. No staff declined to participate in the study.

We interviewed a total of six patients, four parents, and 15 staff members. Table 2 summarizes the characteristics of the participants. Each interview lasted 30–70 minutes and was recorded digitally and transcribed, after removing identifying information. An independent researcher extracted key points and themes from each transcription to create a one-page summary for each interview. These summaries were then imported into NVivo 10 (QSR International) for analysis. The initial data analysis was performed by the first author, under the supervision of the last author (a senior qualitative researcher). The data were coded under the headings of: Strengths, Weaknesses, Opportunities, Threats and Emerging Challenges. The results of the interviews were reviewed by the research team and members of the Transition Committee to ensure that all important ideas were faithfully reported and to ensure the trustworthiness of the coding process.

**Table 2:** **Characteristics of the study participants**

**Service users (n =10)**		
Transition status of the patient	Patients (n =6)	Parents (n =4)
Transitioned	2	2
Not transitioned	1	2
Transitioned but maintained a link with the hospital because of involvement in ongoing research	3	0
**Staff (n =15)**		
	Front-line staff (n =12)	Administrative positions (n =3)
Performance manager		2
Other coordinator		1
Nurse	2	
Occupational therapist	1	
Physiotherapists	2	
Social worker	1	
Dietician	1	
Psychologist	1	
Physician (pediatrician or surgeon)	4	

The interview data were complemented by documents and observations. Documents included pamphlets about the transition period, forms and minutes of meetings from the Transition Committee, and guidelines for the implementation of the transition program. The first author participated in two meetings with the SHC Transition Committee to observe and record discussions with patients preparing to enter the transition process. An initial analysis of the exit surveys has been reported elsewhere [20]. Implementation gaps and areas for improvement were identified by comparing the current transition activities with the actual transition program plan, and with previously published best practices on transitional care.

We carried out a SWOT (Strengths, Weaknesses, Opportunities and Threats) analysis [21]. “Strengths” were defined as advantages associated with the program’s internal organization; “Weaknesses” were defined as limitations associated with the program’s internal organization that might hinder the program’s success; “Opportunities” were defined as external environmental factors that could enhance the success of the transition program; and “Threats” were defined as any environmental factor that could act as a barrier to the transition program. The results of this analysis were cross-validated with information from documents and observations.

Direct quotes have not been used in this report to maintain participant confidentiality. Because of the sensitive nature of the findings and the fact that participants played multiple roles in the project, we chose not to compare results among patients, parents, allied health professionals and staff from adult hospitals, researchers, and members of the Transition Committee. We used the results as the basis for making recommendations on practical strategic directions for the delivery of transition between pediatric and adult care in patients with OI. This study formed part of a broad, comprehensive research project examining quality of care for people living with OI. This project received ethical approval from the McGill University Institutional Research Board.

## Results

### Strengths

#### A well-designed transition model with a monitoring system and an inter-professional team

The key informants reported four main strengths of the transition program that supported a smooth and successful transition. First, the program was closely linked to care coordination and led by an inter-professional team. The key informants including the patient/parent saw this as the most effective means of obtaining continuity of care, which was one of the program’s objectives. Second, the transition program was based on a comprehensive definition of transition that included not only the transfer from pediatric to adult medical care, but also transitioning from school to work, and from dependent to independent living. The involvement of several professionals reflected the program’s goal to provide comprehensive transition support. Third, the transition program allowed time to assess patient readiness. In contrast to many other pediatric hospitals where care stops at 18 years, the SHC’s transition program followed patients until age 21 years. The key informants thought that the age range of 14–21 years should be preserved because it allowed for additional support and ensured a smoother transition. Fourth, the transition program has been monitored and evaluation data on the transition process are therefore available. The SHC systematically carried out transition exit surveys and updated data on the transition process, such as patient referrals to the Transition Committee, were available on its Intranet.

#### Complete organizational support

The transition program received organizational support and commitment from a range of stakeholders, such as the Board of Governors of SHC, front-line staff from a wide range of disciplines, and hospital managers. This support was evident in the early stages of implementing the program at the SHC. For example, there was close and regular supervision of the process via teleconferencing. In interviews, key informants emphasized the strong commitment of hospital managers, which translated into the recruitment of a dedicated nurse coordinator for the transition program and in evaluative research on transition programming.

### Weaknesses

#### Successive organizational changes and discontinuation of certain transition activities

The key informants reported that the Transition Committee overseeing the program had gone through a number of changes resulting in a reduced focus on transition services. The initial committee members who were instrumental in the program’s implementation and early activities had left the committee, and the new committee lacked the same organizational strength. It was felt that there was an inadequate transition and preparation period, and the new committee was therefore less personally connected to the program and the transition process, and consequently did not participate to the same extent as the previous group in committee meetings.

Two other factors contributed to a change of focus and motivation. First, key informants reported a gradual shift in the hospital mission concerning OI patients. Informants perceived that, as caring for people with OI became possible in more local healthcare institutions, the SHC became more a center for confirming an initial diagnosis or for a second opinion, rather than as a center for general care. It was also perceived that the transition needs of patients varied according to their motivation for attending the SHC, and an updated transition-need assessment had not been carried out. The utilization pattern of the SHC transition program by patients had thus not been adjusted since its implementation. Furthermore, the implementation of a new health information system in 2009 was perceived to draw attention and organizational resources away from the transition program.

#### Potential conflict between the transition program and research participation

Our analysis identified a potential conflict between the transition program and patients’ participation in research. Patients with OI are expected to start preparing for transition at age 14; however, research programs may recruit patients and thus extend contact between patients and the SHC beyond the age of 21 years. The Transition Committee therefore sometimes delayed the preparation for transfer to adult care in these research-recruited patients. Documentary analysis indicated that 41% of the 231 patients aged 14–21 years in 2013 were participating in ongoing research. Of the 30 patients aged 21 years who should have completed transition in 2013, 17 (57%) were recruited for ongoing research, and only four had met with the Transition Committee. The remaining 26 patients had not been contacted and their transition process was delayed. Some patients reported feeling that this was unfair, because it meant that some could continue attending the SHC for care while others could not.

#### Gaps in implementation

Our data identified several gaps in the implementation of the transition program. Contrary to the program plan, data on patient satisfaction surrounding the transition process collected at exit survey were not utilized, and collection was eventually stopped. Moreover, there were insufficient tools for patient information, education, and preparation for the transition, with the main source of information being a single pamphlet. Patients were supposed to be approached systematically by the Transition Committee at the age of 14, though several parents noted that the topic was not discussed at follow-up visits and they were concerned that their children would be unprepared for transition without their personal advocacy to contact the committee on their behalf. Furthermore, social activities including camps and transitional events were stopped, in contravention of the program plan. Last, the program did not make arrangements to support staff during the process of “letting go” of patients who they had been following for years.

### Opportunities

#### Multisite transition model with cross-site personnel

Key informants envisioned setting up partnerships with several adult healthcare institutions to scale up the transition program and support all youths with OI. They believed that a multi-site transition program would be more effective, by bridging the divide between adult and pediatric hospitals. The SHC in Montreal employed two neurosurgeons who were responsible for both pediatric and adult patients, as well as a nurse practitioner providing primary care to patients in adult healthcare institutions, who could both thus be considered as cross-site personal. The informants felt that the hospital should capitalize on these cross-site links to improve the reach and effectiveness of the transition program. These professionals would be familiar to transitioning patients and could facilitate contacts with the new adult care environment.

Our study period coincided with the construction of a new hospital, to which the SHC planned to relocate. The new site aimed to provide both pediatric and adult care. The informants saw this as a unique opportunity to renegotiate partnerships between the two care systems and thereby improve the transition program.

#### Users’ evaluation of the program, including second-generation patients

The transition program evaluation plan called for formal feedback from a representative sample of transitioning patients and former patients. Former patients brought their own children with OI to the SHC, and as of July 2013, the SHC had 10 patients whose parents had also been followed at the SHC. The key informants considered this to be a unique opportunity for improving the transition program based on the experiences of former users.

### Threats

#### Dissatisfaction of the care team at the adult care hospital

Members of the care team at the adult hospital reported that young adult OI patients can be demanding and ill-prepared for the reality of the transition to the adult world. They reported frustration over patient comparisons between adult and pediatric hospital functioning, and over reminding patients about appointment times and dealing with cancellations, which tasks were previously managed by parents or a medical secretary in the pediatric setting. Adult care-team members reported that some young adult OI patients were unaware of the differences between the adult and pediatric environments, such as having a smaller team of professionals responsible for their care, and having appointments spread over several days rather than all on the same day, as at the SHC. Furthermore, patients built relationships with their healthcare providers at the SHC over a period of 15–20 years, while they were new patients in the adult care hospital, where they also had to “compete” with other patients, some of whom had life-threatening conditions.

Our data also highlighted systemic barriers that delayed the administration of care to transitioning OI patients. Some key informants mentioned a failure to transfer electronic patient records, which slowed information flow between the two institutions. Finally, key informants pointed out that only a very small number of health professionals outside the SHC had an interest in OI, especially in young adults.

#### Dissatisfaction of former patients

Former patients said that they understood the need to move to an adult care hospital, but would have preferred to stay at the pediatric hospital. They reported difficulties in adapting to the changes demanded by the transition, and noticed that their pattern of service utilization changed. For example, they previously attended the pediatric hospital for regular bone-density check-ups, but only sought care for emergencies as adults, because the adult care hospitals did not offer such check-ups. Some former patients wanted better access to health professionals such as psychologists and geneticists, and some expressed disappointment that their options and risks for child-bearing were not discussed earlier. Despite these barriers, some patients reported feeling fairly well prepared for the transition process, mentioning good parental support as a key factor.

## Discussion

The strengths of the SHC’s transition program for patients with OI included its solid theoretical approach based on a partnership with parents, and a comprehensive model of transition based on fostering independent living and professional integration. Previous reviews and guidelines have noted that these fundamental components are key to achieving a successful transition [22]. Although the transition program benefited from full organizational support, our study revealed a weak organizational capacity to sustain the transition program during successive organizational changes. Strong leadership and priority setting could help steer the transition program through this and other potential changes [23]. Appointment of a “skilled leader” responsible for ensuring continued organizational support and adjusting to changes within the organization would facilitate this process and ensure that focus on the transition program was maintained [22,24]. Appointing a transition coordinator has proven successful in transitional care [25], but our study suggests that a strong, skilled leader would be particularly beneficial. Future transition programs should therefore consider appointing a pediatric nurse practitioner to this role, given their skills as a clinical expert, consultant, change agent, leader, evaluator and educator [26].

Transition programs require sustainable funding. In the SHC setting, such funding depended on philanthropic donors, and lack of such funding resulted in the discontinuation of SHC involvement in transition camps. In general, transition programs require higher levels of funding and staffing that could be best achieved by their integration into routine healthcare, allowing them access to operating funds [27]. In addition, our results highlighted the need to develop more tools for educating patients with OI and for evaluating their readiness for transition. Handouts, posters, booklets and readiness checklists could be developed to complement the currently-available pamphlets and contact information cards. The involvement of families, particularly siblings, could also enhance a smooth transition [28]. Transition programs need to take advantage of new information technologies, for example through the development of online self-management programs and educational activities. Such approaches could also be used to prepare healthcare professionals for the “detachment” process and increase their capacity to “let their patients go” [29]. The use of multiple interventions presents challenges associated with the participation of many informants, including medical schools. However, immediate cost-neutral strategies, such as increasing the level of communication within pediatric and adult institutions, are feasible. For example, the SHC could implement a system to monitor medical-record sharing. It could also consider the option of using electronic patient records, which could promote information exchange, though this could also hinder self-efficacy by taking the responsibility for the task away from the patients. Irrespective of the system used, the transition checklist needs to have an indicator for the successful transfer of medical records [30].

Although data were cross-validated among interviews, observations, and documents, the qualitative findings were limited by the small number of interviewees. In addition, in the specific context of this evaluation, many key informants knew each other, which may have limited their willingness to identify the strengths and weaknesses of the transition program. To minimize this, we chose an interviewer who was not a member of the care team and who encouraged participants to express their views freely. We also minimized subjectivity that might have influenced the results and interpretations of the SWOT analysis by ensuring that all important groups were consulted (patients, parents, health care professionals and hospital managers). Furthermore, the analysis was performed by researchers with diverse backgrounds (clinical, public health, social science, and nursing). Finally, relatively few opportunities and strengths were identified by the participants. This might be seen as a limitation of the SWOT methodology, because they are linked to the external environment of their care system and participants may not have been aware of potential factors [31]. Further longitudinal research could be carried out to investigate the extents of family and staff experiences within the transition program. Despite these limits, the findings of this study may help to inform ongoing practices in other settings. For example, this evaluation confirmed that well-designed transition programs need to evolve and adjust to both the patients’ and organization’s needs. Maintaining an adequate focus on transition might thus require recurrent patient-need assessments and continuous quality-improvement processes. Both of these could be achieved by moving current transition programs beyond the inter-professional focus to include effective partnerships with families, to ensure that the needs of families are updated and accounted for [32,33]. This study also confirmed the usefulness of a dedicated resource to monitor transition programs through evaluative research [27]. Although we demonstrated the need for more educational channels for patients in our institution, we were unable to assess the effectiveness of the currently-available information. Overall, transition programs need standard operating procedures to harmonize the process for the staff.

## Conclusions

This study confirmed that a “one-size-fits-all” transition model for patients with OI was inappropriate across and within institutions. Ongoing transition programs should consider opportunities to create theoretically-sound, tailored transition programs that reflect patient preferences, especially those of young adults with complex and chronic health conditions. In the case of our institution, alignment with other organizational activities should be reviewed, and ongoing evaluation aimed at improving transition programming might be required. The current SWOT analysis and utilization-focused evaluation has led to the development of a comprehensive project to improve the transition program for patients with OI and other conditions requiring special follow-up. This new project will include longitudinal evaluation of the outcomes of the transition program, new transition-preparation activities for patients and their families, and interventions targeted at health professionals. By adopting an integrated knowledge transition approach, the new project will aim to reduce the gap between research and practice, and ultimately ensure that young people with OI can adapt more easily to the adult world.

## Abbreviations

OI: Osteogenesis imperfecta
SWOT: Strengths, weaknesses, opportunities, threats
SHC: Shriners hospital for children
